# Case Report: A dual challenge: navigating cardiac leiomyosarcoma and benign pulmonary mass

**DOI:** 10.3389/fcvm.2025.1572673

**Published:** 2025-06-10

**Authors:** Song Wu, Qiulin Chen, Yi Yang, Jialiang Liu, Yuankun Li, Shengjun Cheng, Yutian Wu

**Affiliations:** Department of Cardiothoracic Surgery, Department of Traditional Chinese Medicine, Chengdu Fifth People’s Hospital, Chengdu, China

**Keywords:** carcinoma, case report, primary leiomyosarcoma, benign tumor, cardiac tumor

## Abstract

Leiomyosarcoma is frequently found in the retroperitoneum, mesentery, omentum, uterus, or subcutaneous tissue. However, primary cardiac leiomyosarcoma is rare and even more uncommon is its coexistence with a benign tumor. We report a case involving a 61-year-old female with a right ventricular outflow tract leiomyosarcoma in conjunction with a benign mass located in the main pulmonary artery. Echocardiography revealed a 2.5 × 2.2 cm isoechoic mass in the right ventricular outflow tract and a 3.8 × 1.8 cm irregular isoechoic mass in the main pulmonary artery. Computed tomography angiography (CTA) indicated a patchy filling defect in a similar location. Initially, pulmonary embolism was considered, however, the possibility of tumors could not be excluded. Given the high risk of mass embolization, we proceeded with emergency surgery. A large, irregular, solid mass was found attached to the wall of the main pulmonary artery, fortunately without involvement of the pulmonary valve. Exploration of the right ventricular outflow tract uncovered an additional solid, smooth, well-encapsulated mass. Immunohistochemical analysis of the right ventricular outflow tract mass confirmed the presence of tumor cells that were positive for Desmin and smooth muscle actin (SMA), while negative for S-100 and myoglobin, leading to a diagnosis of leiomyosarcoma. For the pulmonary mass, Immunohistochemistry revealed the proliferation of fibrous tissue, mucus degeneration, and calcification within the focal area. The imaging characteristics of cardiac leiomyosarcoma combined with benign pulmonary artery tumors may be misinterpreted as thrombosis, however, surgical resection remains a viable treatment option.

## Introduction

Primary tumors of the heart are rare, constituting less than 0.3% of all cases, with approximately 75% classified as benign and 25% as malignant (1). Primary cardiac sarcomas are particularly uncommon, exhibiting an autopsy incidence that ranges from 0.001% to 0.03% (2). Among malignant primary tumors, angiosarcoma is the most prevalent, while leiomyosarcoma is the least common. The anatomical locations of these malignant tumors vary. Angiosarcoma typically arises in the right atrium, whereas osteosarcoma and leiomyosarcoma originate from the left atrium. Notably, to the best of our knowledge, there have been no documented cases in the existing literature of cardiac leiomyosarcoma occurring in conjunction with benign tumors. Here, we present a rare case of primary leiomyosarcoma originating from the right ventricular outflow tract, which is concurrently associated with a benign tumor located in the main pulmonary artery.

## Case presentation

**Figure 1:**
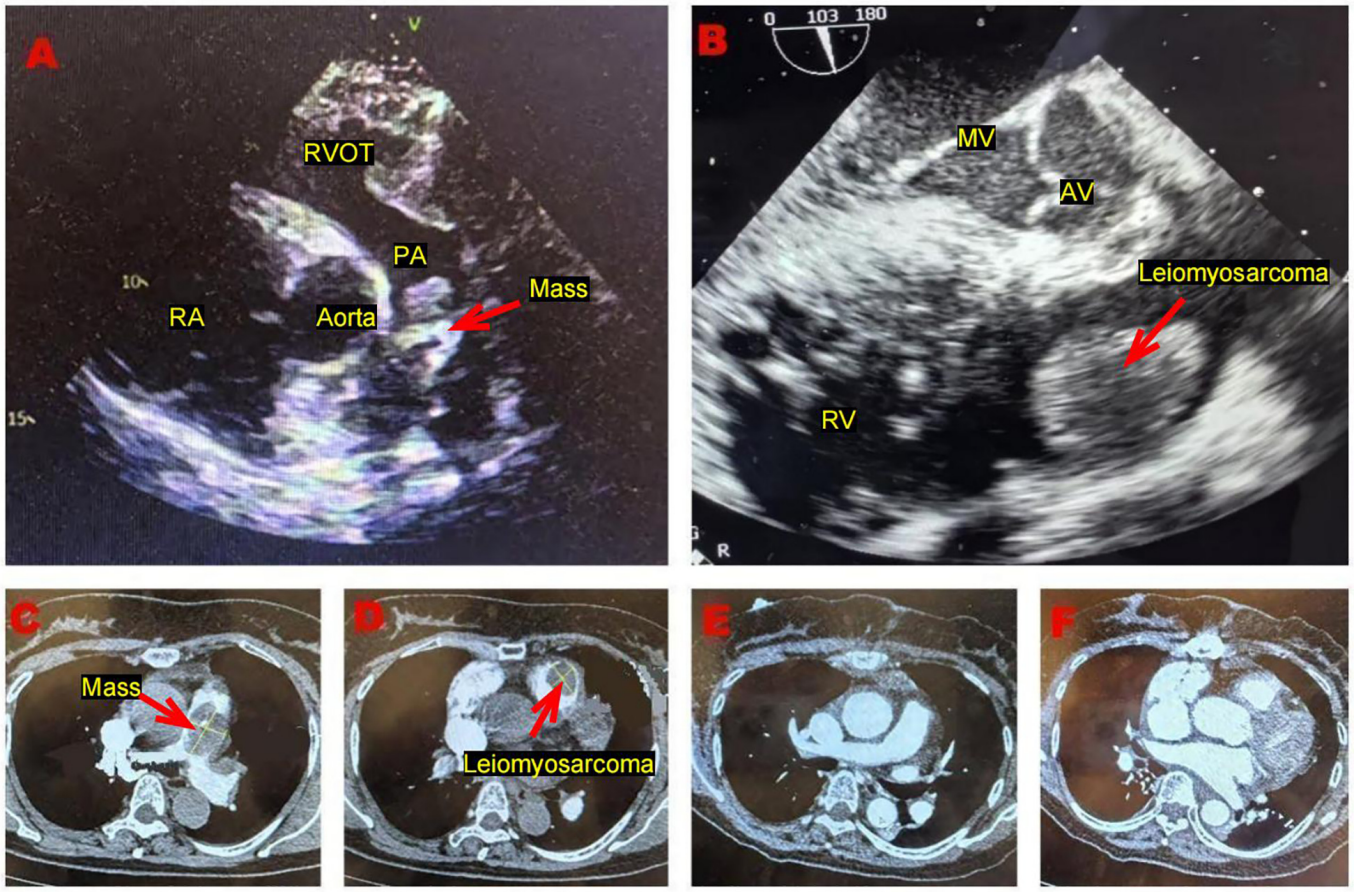
**(A)** Transthoracic echocardiography, performed in the short-axis view of the aorta, demonstrates an irregular isoechoic mass, situated within the main pulmonary artery. **(B)** Transesophageal ultrasound reveals an isoechoic mass with a regular shape situated in the atypical region of the right ventricular outflow tract. This mass appears to be connected to the anterior wall of the right ventricle via a pedicle. **(C)** Pulmonary artery computed tomography angiography reveals a patchy filling defect in the main pulmonary artery. **(D)** Pulmonary artery computed tomography angiography reveals a filling defect in the right ventricle outflow tract. **(E,F)** The postoperative pulmonary artery computed tomography angiography indicates no signs of residual tumor.

A 61-year-old female presented to our hospital with dyspnea and eyelid edema. Approximately three months prior, she experienced similar symptoms at a regional medical facility, where a computed tomography (CT) scan revealed a pulmonary embolism. An attempt at interventional thrombectomy was made, but it was ultimately unsuccessful. Nevertheless, the patient's symptoms showed significant improvement compared to their initial presentation. Following the procedure, she was started on oral anticoagulation therapy with rivaroxaban tablets. Five days before admission, the patient again experienced shortness of breath and notable eyelid edema after physical activity, leading her to seek medical attention at our hospital. Upon admission, a physical examination indicated a body temperature of 36.5°C, blood pressure of 138/79 mmHg, pulse rate of 70 beats per minute, respiration rate of 20 breaths per minute, blood oxygen saturation of 99%, and no murmurs detected during cardiac auscultation. Additionally, a lower limb vascular ultrasound revealed no signs of thrombosis. The pulmonary artery CT angiography (CTA) revealed a patchy filling defect in the main pulmonary artery, measuring approximately 4.8 × 2.7 cm, indicative of a significant area of embolism. Additionally, a filling defect of 2.5 × 2.0 cm was identified in the right ventricle, raising concerns for thrombosis. Echocardiography (Figure 1) demonstrated an isoechoic mass approximately 2.5 × 2.2 cm in size located in the right ventricular outflow tract, alongside an irregular isoechoic mass measuring around 3.8 × 1.8 cm in the pulmonary artery (Figure 1A). A cord-like hyperechoic mass was observed within the latter, oscillating in conjunction with the cardiac cycle. Color doppler flow imaging (CDFI) revealed a filling defect in the pulmonary artery, accompanied by a high systolic peak velocity of 2.8 m/s and severe tricuspid regurgitation. The estimated pulmonary artery pressure was approximately 31 mmHg, and the electrocardiogram displayed sinus rhythm. In our laboratory testing, we measured D Dimer levels at 1.39 µg/ml (0.00–1.00 ug/ml) and recorded brain natriuretic peptide (BNP) levels at 5,524.54 pg/ml (0–120 pg/ml). Blood gas analysis indicated an oxygen partial pressure of 118 mmHg. Initially, we considered the potential presence of a tumor, which could not be ruled out. Consequently, we conducted a tumor marker examination, revealing CA199 levels of 42.73 U/ml (0.00–35.0 U/ml) and CA125 levels of 52.94 U/ml (0.00–30.0 U/ml). Given the critical risk of thrombus detachment at any moment, we proceeded with emergency surgery.

**Figure 2:**
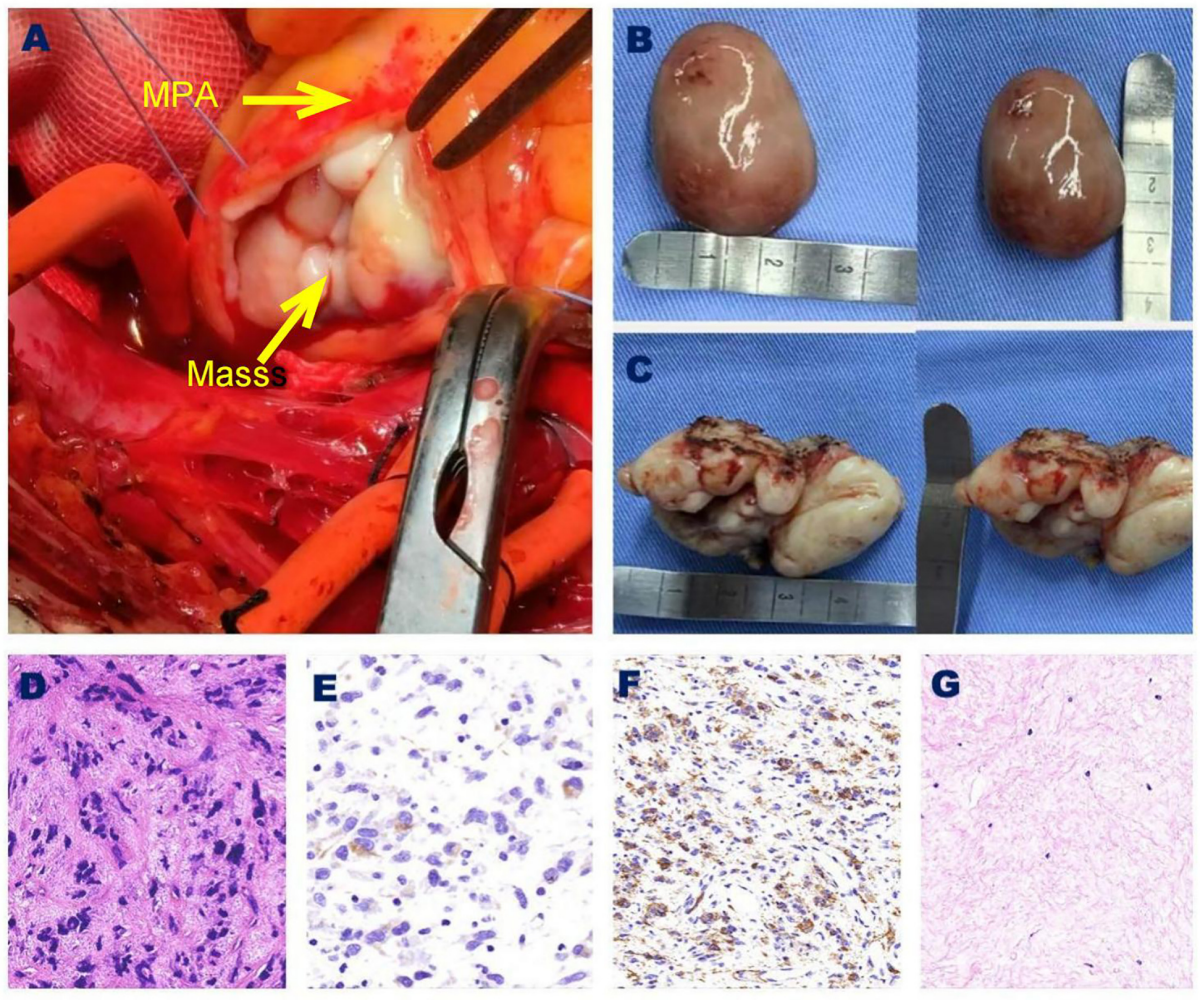
**(A)** The benign tumor in the MPA. **(B)** Right ventricular outflow tract leiomyosarcoma specimen. **(C)** MPA benign tumor specimen. **(D)** Under microscopic examination, leiomyosarcoma is characterized by a high density of tumor cells, primarily consisting of short spindle-shaped cells with a slightly red and eosnophilic cytoplasma. The nuclei of these cells are rounded with blunt ends, exhibiting moderate cellular atypia, and mitotic figures are easily identifiable. (Hematoxylin and Eosin, 40×). **(E)** Immunohistochemical analysis revealed that Desmin was positively expressed in the cytoplasma of tumor cells. (40×). **(F)** Immunohistochemical analysis revealed that SMA was positively expressed in the cytoplasma of tumor cells. (40×). **(G)** Under microscopic examination, pulmonary artery tumors display red-stained proliferated fibrous tissues, alterations in mucus structure, focal calcifications, and a scattered infiltrations of plasma cells. (Hematoxylin and Eosin, 20×). MPA, main pulmonary artery; SMA, smooth muscles actin.

The surgery was performed using extracorporeal circulation. To reduce the risk of detachment and embolism of space-occupying lesions located at the distal end of the pulmonary artery, we first freed and ligated the left and right pulmonary artery trunks. Upon longitudinally incising the pulmonary artery trunk (Figure 2A), we discovered a yellow mass measuring approximately 5.0 × 2.5 cm (Figure 2C), characterized by a hard texture, irregular surface, and narrow base. Fortunately, this mass did not invade the pulmonary valve. Following the complete resection of this mass, we examined the right ventricular outflow tract, where we identified a red mass measuring approximately 2.5 × 3.5 cm (Figure 2B), which also exhibited a hard texture, an intact capsule, and a smooth surface with a narrow base. We successfully performed a complete resection of this mass and continued our exploration, confirming the absence of any residual tumor. Although severe tricuspid regurgitation was noted on preoperative echocardiography, we did not observe any abnormalities in the leaflets or subvalvular structures of the tricuspid valve during the operation, nor did we find significant dilation of the valve annulus. Water injection experiments were conducted to confirm that the tricuspid regurgitation was mild. Therefore, we opted not to treat the tricuspid valve. In the case of the right ventricular outflow tract mass, immunohistochemistry (Figures 2D–F) confirmed the presence of tumor cells that exhibited positive staining for Desmin and SMA, while showing negative staining for S-100 and myoglobin, leading to a diagnosis of leiomyosarcoma. For the pulmonary mass, Immunohistochemistry (Figure 2G) revealed the proliferation of fibrous tissue, mucus degeneration, and calcification within the focal area.

## Discussion

Primary tumors of the heart are infrequent and predominantly benign, with only approximately 25% classified as malignant (3). Among primary malignant cardiac tumors, sarcomas are the most common, with angiosarcoma identified as the principal subtype (4). Leiomyosarcoma, a rare form of cancer, originates from smooth muscle cells and is typically found in locations such as the retroperitoneum, mesentery, omentum, uterus, or subcutaneous tissue (5). With an incidence rate of less than 0.25% among all cardiac tumors, primary cardiac leiomyosarcoma is an exceptionally rare condition, first documented by Nakashima et al. in 1941 (6).

Cardiac leiomyosarcomas are highly aggressive tumors associated with a poor prognosis, and they are more frequently found in the left atrium (7). Numerous case reports (8–12) have documented the potential for leiomyosarcoma to arise in various cardiac structures, including the pulmonary valve, left ventricle, right atrium, and right ventricle. In this case, the patient was diagnosed with primary cardiac leiomyosarcoma located in the right ventricular outflow tract. Although previous literature (13–17) has documented isolated cases of tumors in similar locations, there have been no reported instances of primary cardiac leiomyosarcoma coexisting with primary benign cardiac tumors. This case represents the first documented instance confirming the rare phenomenon of coexistence between two primary cardiac tumors. Epidemiological data (18) suggest that cardiac leiomyosarcomas typically manifest around the age of 40, exhibit no gender preference, and symptoms generally do not present until the advanced stages of the disease.

The clinical symptoms and signs of cardiac tumors vary depending on the tumor's location and size. Common clinical manifestations of leiomyosarcoma include dyspnea, pericardial effusion, chest pain, atrial arrhythmias, and heart failure. The patient was admitted to the hospital for the treatment of dyspnea and eyelid edema. Following the surgical resection of the tumor, the patient's dyspnea improved significantly, and the eyelid edema subsided. This suggests a potential correlation between eyelid edema in patients and the presence of tumors. From the perspective of right heart failure, although BNP levels are significantly elevated, the clinical manifestations do not exhibit the typical features of right heart failure. Specifically, there is an absence of lower limb edema, liver congestion or enlargement, and a negative hepatic jugular venous return sign. Additionally, cardiac ultrasound did not reveal a significant decrease in right heart systolic and diastolic function. Therefore, we excluded right heart failure as a contributing factor and speculated that the primary reason for the patient's elevated BNP levels was the tumor occupying the ventricular cavity. This condition likely led to an increase in right ventricular filling pressure and ventricular wall tension, which, in turn, stimulated the synthesis and release of BNP by myocardial cells. The analysis of tumor-related mechanisms indicates that tumor cells can secrete various tumor markers, including cancer antigens, alpha-fetoprotein, and tissue polypeptide antigens. These substances may provoke immune responses in the body and theoretically have the potential to trigger inflammatory allergic reactions. However, the patient exhibited only eyelid edema without typical inflammatory allergic symptoms such as localized redness, swelling, or itching. Consequently, this factor is excluded from consideration. Given the patient's elevated right heart pressure, it is plausible that there are pathological and physiological changes associated with venous reflux obstruction. Therefore, it is crucial to maintain a high level of vigilance regarding the diagnosis of superior vena cava syndrome. Superior vena cava syndrome is characterized by partial or complete obstruction of blood flow from the superior vena cava to the right atrium, resulting from various etiologies. This obstruction leads to increased venous pressure and compensatory dilation of the distal veins. Typical clinical manifestations include edema of the head, neck, face, and upper limbs, as well as difficulty breathing, cerebral edema, and cyanosis of the lips and skin. Common causes of this syndrome encompass malignant tumors (such as lung cancer, thymic cancer, and lymphoma), venous thrombosis, thyroid enlargement, detachment or infection of pacemaker electrodes, among other factors. Previous research (19) has indicated that superior vena cava syndrome can present as a unique clinical manifestation of simple bilateral eyelid edema. The mechanism underlying this phenomenon primarily involves two key aspects. First, there exists an extensive network of communication branches between the ophthalmic vein and the superior vena cava system. When the pressure in the superior vena cava significantly increases, the pressure in the ophthalmic vein correspondingly rises through these communication branches. This pressure increase promotes fluid filtration across the vascular wall, leading to leakage into the tissue interstitial space, which results in eyelid edema. Second, superior vena cava syndrome can induce local tissue hypoperfusion, which contributes to hypoxia and metabolic dysfunction. This, in turn, causes endothelial cell damage and a significant increase in vascular wall permeability. Consequently, plasma components, including liquids and proteins, seep out of the blood vessels and accumulate in large quantities within the interstitial spaces of the eyelid tissue, further exacerbating the degree of eyelid edema. During the diagnosis and treatment process for this case, the patient's central venous pressure was not monitored prior to surgery, preventing verification of our hypothesis. This oversight represents one of the limitations of our study.

Cardiac leiomyosarcoma is predominantly diagnosed through imaging examinations, with cardiac ultrasound being a widely utilized diagnostic tool. Existing literature (20) indicates that the accuracy of cardiac ultrasound in identifying cardiac leiomyosarcoma can reach up to 80%. In this particular case, the patient was misdiagnosed as having a thrombus based on preoperative transthoracic ultrasound for several reasons. Firstly, although no obvious thrombus was detected during the patient's lower extremity venous ultrasound, the widening of the inner diameter of the intermuscular vein does not eliminate the possibility of thrombosis. Secondly, the mass was observed occupying the pulmonary artery, where the irregular shape of the space-occupying lesions fluctuated with the cardiac cycle, mimicking the sonographic appearance of a partially organized thrombus. In contrast, the space-occupying lesions located in the right ventricular outflow tract displayed a regular shape, suggesting a higher likelihood of benign lesions. Finally, an intravascular filling defect was identified on the patient's pulmonary artery CTA. Given the limitations of transthoracic ultrasonography and the slight elevation in the patient's tumor markers, we initially considered the possibility of a benign cardiac tumor, such as myxoma. The literature (21) indicates that cardiac magnetic resonance imaging (MRI) provides superior visualization of the overall characteristics of soft tissue compared to computed tomography (CT). However, the examination duration is longer. Given the principle of prioritizing patient safety and the necessity to avoid the risk of embolism during the examination process, we must forgo this imaging modality. Consequently, we proceeded with emergency surgery. During cardiopulmonary bypass, we routinely perform esophageal ultrasound, a technique that enables visualization of echogenic masses, such as the right ventricular outflow tract, which typically exhibit regular shapes. A notable pedicle can be observed attached to the anterior wall of the right ventricle (Figure 1B). In contrast, mixed echogenic masses located in the pulmonary artery often display irregular shapes and may indicate the presence of tumors. This observation underscores the existence of bigenic tumors of the heart. When tumors are situated in atypical locations, a cautious diagnostic approach is warranted. Esophageal ultrasound has demonstrated higher sensitivity in assessing cardiac tumors. While pulmonary artery CTA is regarded as the gold standard for diagnosing pulmonary embolism (22), thrombi typically present as filling defects under contrast medium. Interestingly, the imaging findings in this patient suggest that the presence of a filling defect in blood vessels or cardiac chambers may also indicate a tumor. Distinguishing between thrombi and neoplastic lesions can be extremely challenging. In this case, the patient was initially diagnosed with cardiac thrombosis at a local medical institution and subsequently underwent interventional thrombectomy and anticoagulation therapy. Although the patient's symptoms improved, the duration of relief was brief. Therefore, for patients with thrombosis-related diagnoses, it is critical to consider the possibility of a tumor when conventional treatment options are not feasible.

The treatment of cardiac leiomyosarcoma involves a variety of approaches, including surgery, radiation therapy, chemotherapy, targeted therapy, and immunotherapy. Currently, surgical tumor resection is the only treatment modality shown to effectively eradicate this disease and improve survival rates (23). Literature (24) indicates that the average survival time for patients who do not undergo surgery ranges from 6 to 12 months, while those who receive surgical intervention have an average survival time of approximately 24 months. Pathological examination confirmed the diagnosis of cardiac leiomyosarcoma in this patient. Postoperative pulmonary artery computed tomography angiography (Figures 1E,F) and cardiac ultrasound revealed unobstructed blood vessels and no abnormalities in the cardiac chambers. Furthermore, a whole-body positron emission tomography-computed tomography (PET-CT) scan was performed to check for metastatic tumors. Fortunately, no tumors were detected in other organs, and no residual tumor was found in the cardiac chamber, resulting in an R0 resection. Currently, the adjuvant treatment options following cardiac leiomyosarcoma surgery remain uncertain. For metastatic or unresectable leiomyosarcoma, the primary treatment regimen remains anthracycline-based chemotherapy, with doxorubicin serving as the most representative agent. Doxorubicin operates by inhibiting topoisomerase II and inducing oxidative stress, which leads to DNA damage. Karavelioglu et al. (25) reported a case involving an 86-year-old female patient diagnosed with epicardial leiomyosarcoma, presenting as cardiac tamponade. She was administered doxorubicin at a weekly dose of 25 mg/m^2^ for five months without surgical intervention, and she continued to survive, suggesting that this treatment was both safe and effective. In contrast, Sachdev et al. (26) noted that the benefits of doxorubicin are limited by dose-dependent adverse effects, particularly cardiotoxicity, which typically occurs when the cumulative dose exceeds 550 mg/m^2^. One proposed mechanism underlying this cardiotoxicity is the conversion of doxorubicin into its active metabolite, doxorubicinol. Therefore, it is essential to pursue the development of novel delivery methods aimed at reducing this toxicity. One such method involves the use of albumin ligands to create a prodrug form of doxorubicin, known as aldoxorubicin. This prodrug consists of the C-13 section of doxorubicin combined with a blend of six equine sources and is synthesized through the reaction between imidocaproic acid and hydrazine. In the bloodstream, the maleimide component forms a covalent bond with Cys34 of albumin. Upon exposure to the acidic environment of tumors, the hydrazone ligand degrades, resulting in the release of doxorubicin. An international, multicenter, phase 2b, open-label, randomized study (27) enrolled 123 patients with untreated locally advanced or unresectable disease, assigning them to either an aldoxorubicin group (*n* = 83) or a doxorubicin group (*n* = 40). The median progression-free survival was significantly greater in the aldoxorubicin group, with a median of 5.6 months (95% CI, 3.0–8.1), compared to 2.7 months (95% CI, 1.6–4.3) in the doxorubicin group (*P* = 0.02). Furthermore, the monthly progression-free survival rate was superior in the aldoxorubicin group, recorded at 46% vs. 23% in the doxorubicin group (*P* = 0.02). The median overall survival was reported as 15.8 months (95% CI, 13.0 to unavailable) for the doxorubicin group and 14.3 months (95% CI, 8.6–20.6) for the aldoxorubicin group (*P* = 0.21). No acute cardiotoxic effects were observed with either treatment. However, three patients receiving doxorubicin exhibited a left ventricular ejection fraction of less than 50%, suggesting that doxorubicin alone may prolong progression without improving survival. Improvements in 6-month progression-free survival and tumor response rates were found to be superior with aldoxorubicin, indicating its promising therapeutic potential. However, since the drug remains in the research stage and the available literature is relatively limited, further clinical studies may be necessary in the future to validate its efficacy. Additionally, there are related reports (28–30) concerning combined drug chemotherapy. Tap et al. (31) evaluated the efficacy of doxorubicin combined with a placebo vs. doxorubicin combined with olaratumab in the treatment of advanced sarcoma through a randomized controlled trial. The conclusion indicated no significant difference in overall survival between the two treatment regimens. Given the potential for cardiac toxicity, we plan to administer low-dose doxorubicin alone for subsequent treatment. The specific regimen will involve 25 mg/m^2^, administered weekly over a course of 4 weeks, for a total of 8 cycles. Throughout the treatment process, we will closely monitor for possible adverse reactions, including cardiotoxicity, bone marrow suppression, and gastrointestinal reactions, adjusting the treatment plan as necessary based on the patient's specific circumstances. We have communicated with the patient multiple times, providing comprehensive medical explanations and psychological counseling; however, the patient has declined to accept subsequent standardized treatment. Consequently, we were unable to obtain follow-up data for further treatment, which poses limitations to this study.

Primary cardiac sarcoma, especially in conjunction with benign pulmonary artery tumors, is a notably rare condition that is frequently misdiagnosed. Surgical resection remains the preferred treatment approach. The choice of postoperative adjuvant therapies requires a multidisciplinary discussion and a collaborative decision-making process.

## Data Availability

The original contributions presented in the study are included in the article/Supplementary Material, further inquiries can be directed to the corresponding author.
